# Effects of super-class cannabis terpenes beta-caryophyllene and alpha-pinene on zebrafish behavioural biomarkers

**DOI:** 10.1038/s41598-022-21552-2

**Published:** 2022-10-14

**Authors:** Andréa Johnson, Alycia Stewart, Ismaeel El-Hakim, Trevor J. Hamilton

**Affiliations:** 1 Department of Psychology, MacEwan University, Edmonton, AB Canada; 2 Neuroscience and Mental Health Institute, University of Alberta, Edmonton, AB Canada

**Keywords:** Drug discovery, Neuroscience, Psychology

## Abstract

Terpenes possess a wide range of medicinal properties and are potential therapeutics for a variety of pathological conditions. This study investigated the acute effects of two cannabis terpenes, β-caryophyllene and α-pinene, on zebrafish locomotion, anxiety-like, and boldness behaviour using the open field exploration and novel object approach tests. β-caryophyllene was administered in 0.02%, 0.2%, 2.0%, and 4% doses. α*-*pinene was administered in 0.01%, 0.02%, and 0.1% doses. As α-pinene is a racemic compound, we also tested its (+) and (−) enantiomers to observe any differential effects. β-caryophyllene had only a sedative effect at the highest dose tested. α-pinene had differing dose-dependent effects on anxiety-like and motor variables. Specifically, (+)-α-pinene and (−)-α-pinene had significant effects on anxiety measures, time spent in the thigmotaxis (outer) or center zone, in the open field test, as well as locomotor variables, swimming velocity and immobility. (+ /−)-α-pinene showed only a small effect on the open field test on immobility at the 0.1% dose. This study demonstrates that α-pinene can have a sedative or anxiolytic effect in zebrafish and may have different medicinal properties when isolated into its (+) or (−) enantiomers.

## Introduction

Cannabis terpenes found in the *Cannabis sativa* plant have emerged as candidate therapeutic compounds^1^ following the potential health benefits of the phytocannabinoids ∆^9^-tetrahydrocannabinol (THC) and cannabidiol (CBD)^2^. Terpenes, a major class of phytochemicals, form the essential oils of plants and flowers and are responsible for their varying aromas, flavours, and colours^1,3–6^. In the cannabis plant, terpenes are found in the glandular trichomes of the inflorescence of the female plant, the same glands that secrete the common phytocannabinoids, THC and CBD^3,5^, and vary within and across the many different strains^4,5,7,8^. There are over fifty cannabis terpenes most commonly found in North American cannabis strains, eight of which predominate to form a “Terpene Super Class”: myrcene, terpinolene, ocimene, limonene, ⍺-pinene, humulene, linalool, and β-caryophyllene^9^.

Terpenes are hydrocarbon compounds that consist of varying numbers of isoprene molecules and are classified according to the number of pairs of isoprenes they are made up of^10^. The most prevalent types of terpenes in cannabis consist of either 2 isoprene molecules (monoterpenes) or 3 isoprene molecules (sesquiterpenes), and less commonly, 4 isoprene molecules (diterpenes)^10^. Monoterpenes are highly volatile and contribute more to the aroma of the cannabis plant, while sesquiterpenes are more stable and less likely to break down during plant processing. Each cannabis strain has a unique terpene profile which contributes to the different psychoactive and medicinal properties of each strain^11^. Recent research has found terpene compounds to have a myriad of potential medicinal properties including, but not limited to, anti-inflammatory, anxiety-reducting (anxiolytic) and antidepressant effects in humans and mice^1,6,12,13^. Two candidate terpenes from the super class with potential therapeutic effects are β-caryophyllene and ⍺-pinene.

β-caryophyllene (βCP), is one of the major sesquiterpenes found in cannabis^3,8,14^ and is also present in clove, rosemary, black pepper, and lavender. To date, studies have shown this compound to have anticancer properties as well as anti-inflammatory properties^12,15,16^. Additionally, Galdino and colleagues^17^ found that mice dosed with βCP displayed anxiolytic behaviour in the elevated plus maze and light dark test. They also found βCP to decrease latency to sleep and increased duration of sleep time. A similar study by Bahi and colleagues^13^ found that mice dosed with βCP also showed reduced anxiety-like behaviour in the elevated plus maze, open field test, and marble-burying test. Mice also demonstrated anti-depressive behaviour in behavioural assays validated for measuring depression, such as the novelty suppressed feeding and tail suspension tests. Machado and colleagues^14^ also demonstrated the anxiolytic effects of βCP on mice in the light/dark test. Rabbani and colleagues^18^ found that a hydroalcoholic extract of βCP (at 150 and 200 mg kg^−1^) showed anxiolytic effects similar to diazepine (at 0.5 mg kg^−1^) on mice in the elevated plus maze. βCP shows promise as an anxiolytic compound, however, there are no studies to date on its effects in zebrafish models.

In addition to the number of pairs of isoprene molecules, terpenes also differ in regard to whether they are monocyclic or bicyclic^19^. Bicyclic terpenes are a set of optical isomers (enantiomers) that are non-superimposable mirror images of each other^19^. Pinene is a bicyclic compound and one of the most prominent cannabis monoterpenes found in nature^3^, most commonly, in lavender, rosemary, and conifers^11,12^. Pinene has two constitutional isomers, α-pinene (αPN) and β-pinene (βPN), each are racemic compounds that are separable into S(+) or R(−) enantiomers^19^. Previous research has shown αPN to have an anxiolytic effect on mice after inhalation of αPN derived from cypress of the genus, *Chamaecyparis obtuse*^20^, and from pine of the genus, *Pinus*^21^. Satou and colleagues^20^ found mice dosed with αPN demonstrated decreased anxiety behaviour in the elevated plus maze, and its effects to be maintained after repeated exposure. Yang and colleagues^21^ also found αPN to enhance sleep duration, quality, and brain wave density by direct binding to GABA_A_ receptors. αPN has also shown to have strong anti-inflammatory and antibiotic properties^3^. Additionally, enantiomers from each pinene compound have different effects^19^: The positive enantiomers, (+)-αPN and (+)-βPN, exhibited significantly higher antimicrobial effects when compared to the negative enantiomers. Some enantiomers can produce opposite behavioural effects, like the ketamine analog, methoxetamine^22^. The extent to which αPN enantiomers may vary in their ability to alter behaviour is unknown.

Zebrafish (*Danio rerio*) are a well-established model for testing neurobiology and drug action. Recently, Murr^7^ demonstrated the anticonvulsant effects of two terpenes commonly found in cannabis, myrcene and limonene, on zebrafish induced with epileptic-like seizures. In an acute dosing experiment, limonene and myrcene were shown to decrease zebrafish anxiety-like behaviour in the open field exploration test while linalool demonstrated a sedative effect on zebrafish locomotion^23^. There are many empirically validated behavioural assays for testing zebrafish anxiety-like behaviour and boldness, which include the open field-exploration test and novel object approach test. The open field exploration test is a commonly used paradigm, adapted from rodent models, that has been validated to measure zebrafish anxiety-like behaviour^24–26^. In this test, anxiety-like behaviour is measured by the amount of time the zebrafish spends in specific zones of the arena. Within the arena are 3 significant zones: the outer zone, known as the thigmotaxis zone, in which a fish may demonstrate anxiety-like (escape or centrophobic) behaviour by hugging the walls of the arena, the transition zone which leads to the center of the arena, and the inner zone or center zone. The duration of time spent in the inner zone can be indicative of exploratory behaviour into the ‘less protected' center of the arena, which is associated with a decrease in anxiety-like behaviour^24^. Along with cumulative duration in arena zones, alterations in locomotion such as swimming velocity and immobility may also be indicative of anxiety-like behaviour. The novel object approach test is another common paradigm among zebrafish models, where an unfamiliar object is placed into the open field testing arena and is used to quantify anxiety-like behaviour by avoidance or boldness^27^. Avoidance is calculated by time spent in the thigmotaxis zone away from the object and is indicative of heightened anxiety due to an unfamiliar object in the arena. Boldness is assessed by calculating the increased time spent in the center zone near the novel object^24^. In a study by Hamilton and colleagues^28^, the administration of ethanol (a common and reliable anxiolytic drug used in animal research) in zebrafish significantly increased the number of approaches to a novel object and cumulative time spent close to the object. As previously mentioned, alterations in locomotor behaviour relative to the introduction of the novel object may also indicate levels of anxiety in this test.

Of the eight super class terpenes, the present study tested the anxiolytic effects of commonly found and currently understudied cannabis terpenes, βCP and αPN along with (+) and (−)-αPN enantiomers of αPN, on zebrafish behaviour in two common behavioural paradigms, the open field exploration test and novel object approach test.

## Method and materials

### Animals and housing

Adult zebrafish (*Danio rerio*) of mixed gender (~ 50:50, male:female) were obtained from MacEwan University’s in-house breeding facility in December of 2020 and February of 2021. Broodstock zebrafish were obtained from the University of Ottawa (Ottawa, ON, Canada). All zebrafish were from a wild-type strain. Zebrafish were housed in 3 L and 10 L polyurethane tanks within an Aquatic Habitats (AHAB, Aquatic Ecosystems, Inc. Apopka, FL, USA) three-tier bench top system. Housing facility water consisted of reverse osmosis water buffered with non-iodized salt, sodium bicarbonate, acetic acid and maintained to a pH of 6.5 to 8.0. Housing facility water was continuously re-circulated and filtered through 50 µm of mechanical and activated carbon, UV irradiated, and maintained at 26 to 30 °C. Zebrafish were on a 12-h light/dark cycle from 8:00 AM to 8:00 PM and were fed once daily with Gemma Micro 300 fish flakes (Gemma Micro, Maine, USA). Fish were not fed on testing days until after experiments were conducted.

### Drug administration

Terpene solutions were made fresh daily by adding each treatment dose to 400 mL of housing facility water. Due to low solubility, terpene solutions were stirred vigorously and left to dissolve for up to 25 min until there were no visible residual oils in the dosing beaker. Solution pH was monitored before and after the addition of terpene compounds and stayed within a pH of 6.8–7.5. The treatment vessel (i.e. dosing beaker) was surrounded by white corrugated plastic to reduce any behavioural alterations due to visual conspecific cues^29^. Individual zebrafish randomly assigned to either a control group or to one of the terpene conditions remained in the solution for 10 min.

#### β-caryophyllene

β-caryophyllene (≥ 80% sum of isomers; sourced from SIGMA, Ontario, Canada), was mixed into a 600 mL dosing beaker containing 400 mL of housing facility water in 0.02 (0.98 μmol; n = 27), 0.2 (9.8 μmol; n = 18), and 2.0% (98.0 μmol; n = 17) doses. The control solution consisted of 400 mL of housing facility water (n = 21). Our starting dose was determined by pilot testing and doses used in a previous terpene study with zebrafish^23^. An additional experimental group was added with 4% (195.7 μmol; n = 19). β-caryophyllene dissolved in 0.1% ethanol (EtOH) in 400 mL of housing facility water to test solubility effects and increase the terpene dose. The control solution for this group was made with 400 mL of housing facility water mixed with 0.1% EtOH (n = 24).

#### (+ /−)-α-pinene

(+ /−)-α-pinene (98%; sourced from Sigma-Aldrich, Ontario, Canada), was mixed into a 600 mL dosing beaker containing 400 mL of housing facility water with 0.01 (0.73 μmol; n = 23), 0.02 (1.5 μmol; n = 24), and 0.1% (7.3 μmol; n = 20) doses. The control solution consisted of 400 mL of housing facility water (n = 32). All pinene doses were based on careful pilot testing and previous murine studies where an oral administration of 10 μL/L (0.01%) of α-pinene was shown to be an effective dose for mice^30^.

#### S(+)-α-pinene

S(+)-α-pinene (≥ 99%; sourced from Sigma-Aldrich) was mixed into a 600 mL dosing beaker containing 400 mL of housing facility water with 0.01 (0.73 μmol; n = 13), 0.02 (1.5 μmol; n = 13), and 0.1% (7.3 μmol; n = 13) doses. The control solution consisted of 400 mL of housing facility water (n = 13).

#### R(−)*-*α-pinene

R(−)-α-pinene (99%; sourced from Sigma-Aldrich) was mixed into a 600 mL dosing beaker containing 400 mL of housing facility water with 0.01 (0.73 μmol; n = 15), 0.02 (1.5 μmol; n = 16) and 0.1% (7.3 μmol; n = 13) doses. The control solution consisted of 400 mL of housing facility water (n = 19).

### Behavioural testing

#### Open field exploration test

**Figure 1:**
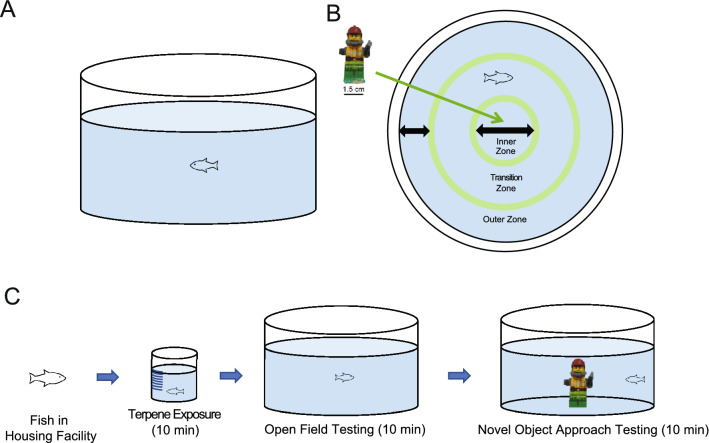
Testing Apparatus. (**A**) The open field testing arena consisted of a white plastic cylinder (26.0 cm in diameter) filled to a water depth of 5.0 cm. (**B**) For the novel object approach test, a multicoloured Lego figurine with a height of 4.25 cm was affixed to the center of the open field arena using a ′1 × 2′ Lego brick. The test arena was partitioned into three zones in EthoVision XT motion tracking software (inner, transition, and outer zones). (**C**) Diagram of experimental procedure: Individual fish were netted from the housing facility into a dosing beaker for 10-min of terpene exposure, then transferred to the open field arena. After a 10-min trial in the open field test, a Lego figurine was placed into the center of the arena for the 10-min novel object approach test trial.

All behavioural testing protocols used in this study were based on a previous study conducted by Szaszkiewicz and colleagues^23^. Experimentally naïve fish were acclimated in the housing facility for a minimum of one week prior to testing. On testing days, zebrafish were transferred by netting into a 3 L polyurethane habituation tank from the housing facility in the testing room. Prior to experimentation, zebrafish were habituated in the testing room for approximately 25 min. Habituation tanks were fully surrounded by white corrugated plastic to reduce exposure to extraneous visual stimuli. After habituation, individual zebrafish were netted into a 600 mL dosing beaker containing either the terpene or control solutions as described above. Control fish were chosen by random selection and interspersed throughout testing days to control for any time-of-day effects. After dosing, individual zebrafish randomly assigned to either a control group or to one of the terpene conditions were immediately netted and placed into the open field testing arena. After 10 min in an open field testing arena, a novel object was then introduced and fish behaviour recorded for an additional 10 min (Fig. 1C).

The open field testing apparatus was a 26 cm circular plastic arena with a height of 11.5 cm and water level filled to 5 cm (Fig. 1A). The testing arena was enclosed by three walls comprised of white corrugated plastic to minimize exposure to extraneous visual stimuli. Fish were individually netted and placed into the testing arena halfway between the center and thigmotaxis zones. Recording of the fish then began and trials lasted 10 min. Proxies used to measure anxiety-like behaviour for the main variables of interest were the cumulative duration of time spent in the thigmotaxis (outer) zone and time spent in the inner zone. Locomotor variables, velocity and immobility, were also assessed. Zones were created within Noldus EthoVision XT software (v. 11.0, Noldus, Wageningen, NL)  and included annular zones consisting of a center zone of 8.6 cm, a transition zone of 4.3 cm, and a thigmotaxic zone of 4.3 cm (Fig. 1B).

#### Novel object approach test

After a duration of 10 min in the open field exploration test, a novel object was added to the middle of the testing arena and swimming behaviour was recorded for another 10 min. The novel object was a multicoloured Lego figurine (2 cm × 4.25 cm; Fig. 1B) affixed to the bottom of the center of the tank by a small 1 × 2 Lego brick. Behaviour was quantified by time spent in arena zones relative to the novel object, in this case the thigmotaxis and center zone, as well as locomotor variables, velocity and immobility. After every fifth or sixth trial the H_2_O in the testing arena would be refreshed to prevent build-up of waste and excess terpene compound, and to maintain water temperature^31^. Once each trial ended, zebrafish were sexed and placed back into a housing tank and fed. The water temperature in the housing tank of experimental zebrafish, drug solution, and testing arenas was kept between 26 and 28 °C with seedling heat mats (Hydrofarm Horticultural Products, Petaluma CA). Luminance in all testing arenas was measured at ~ 32 8 cd/m^3^ (cal SPOT photometer; Cooke Corp. CA, USA). A Basler GenICam acA1300-60gc Area Scan video camera (Basler Inc., USA) was suspended approximately 1 m above testing arenas to record zebrafish behaviour. Zebrafish movement was tracked and recorded using EthoVision XT tracking software. Researchers were not blinded to treatment, however, all fish were tested in an identical manner and analyzed using a motion-tracking software system. Immobility was determined at a 5% threshold, whereby, a fish would be considered immobile if tracking software detected less than a 5% change in the pixels of the body of the fish^23^.

### Statistical analysis

All data were analyzed using GraphPad Prism Software (Version 9.1.2; GraphPad, San Diego, CA, USA). Data were assessed for normality using the D’Agostino-Pearson omnibus normality test and Bartlett’s test for equality of variances. Parametric data was analyzed using an ordinary one-way ANOVA followed by post-hoc Dunnett’s multiple comparison test. Non-parametric data was analyzed using a Kruskal–Wallis with post-hoc Dunn’s multiple comparison test. The Brown-Forsythe ANOVA was used for data with unequal variance. An alpha level of *p* < 0.05 and a 95% confidence interval was used to indicate statistical significance. All values are presented as mean ± standard error in measurement (S.E.M.). Data were omitted for fish in treatment groups that reacted with heightened sensitivity and displayed extreme sedation and locomotor impairment during testing. Data were also excluded from analyses if the full data was not acquired by tracking software for the total time each fish spent in the arena. This resulted in the following number of fish removed per condition: 0% (+ /−)-αPN group (n = 2), 0.01% (+ /−)-αPN group (n = 1), 0.02% (+ /−)-αPN group (n = 1), 0% (−)-αPN group (n = 6), 0.01% (−)-αPN group (n = 7), 0.02% (−)-αPN group (n = 4), 0.1% (−)-αPN group (n = 4), 0% (+)-αPN group (n = 7), 0.01% (+)-αPN (n = 10), 0.1% (+)-αPN group (n = 2), 0% βCP group (n = 4), 0.02% βCP group (n = 3), 2.0% βCP group (n = 4), 4.0% βCP group (n = 4). These fish were not included in the sample sizes noted in 2.2. In the βCP experiment, data from the control group and 0.1% EtOH (used as a vehicle control for 4.0%), were compared and no significant differences in fish behaviour were found so control groups were combined.

### Ethics statement

All experiments were approved by the MacEwan University Animal Ethics Board (AREB) under protocol number 101853 in compliance with the Canadian Council for Animal Care (CCAC) experimental guidelines. All authors complied with ARRIVE guidelines.

## Results

### Effects of (+ /−)*-*α-pinene in the open field exploration test

**Figure 2:**
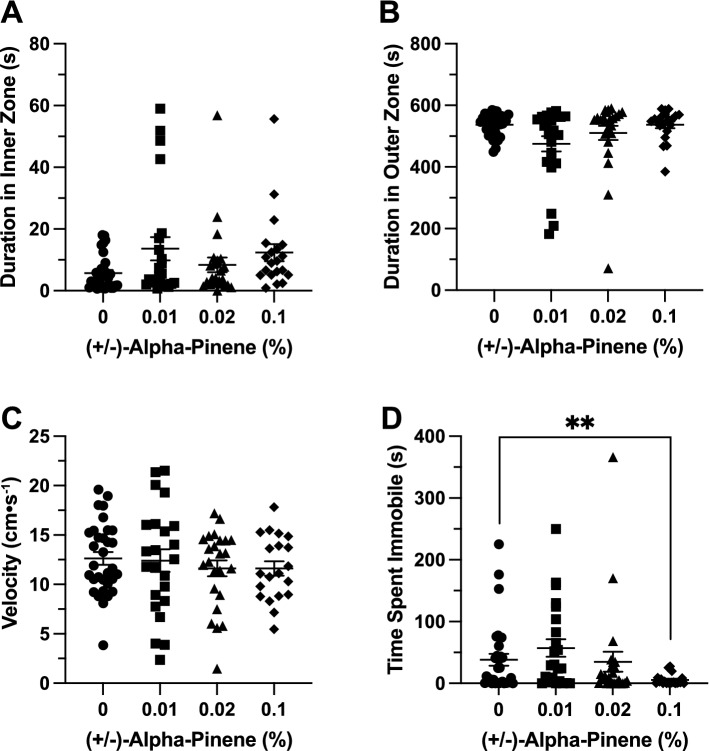
The effects of (+ /−)-alpha-pinene administration in the open field test. Average duration of time fish spent in the (**A**) inner and (**B**) outer, ‘thigmotaxis’ zone during the open field test. Fish locomotion was quantified in the open field test by measuring (**C**) swimming velocity and (**D**) time spent immobile. All data are presented as mean ± S.E.M. Significant differences between controls and (+ /−)-alpha-pinene treated groups are indicated by **(*p* < 0.05).

*Time in Zones*. (+ /−)-αPN did not have a significant effect on duration of time spent in the inner zone between groups (F(3, 59.97) = 2.061, *p* = 0.115; Fig. 2A). (+ /−)-αPN did not have a significant effect on duration of time spent in the thigmotaxis zone between groups (F(3, 56.39) = 2.679, *p* = 0.056; Fig. 2B).

*Locomotion*. (+ /−)-αPN did not have a significant effect on velocity between groups (F(3, 95) = 0.4171, *p* = 0.741; Fig. 2C). (+ /−)-αPN did have a significant effect on duration of time spent immobile between groups (F(3, 64.29) = 2.780, *p* = 0.048). A post-hoc analysis using Dunnett’s multiple comparisons test found a significant decrease in time spent immobile in the 0.1% group (5.6 ± 1.9 s, n = 20, *p* = 0.008) when compared to the control group (38.1 ± 9.8 s, n = 32; Fig. 2D).

### Effects of (+ /−)-α-pinene in the novel object approach test

**Figure 3:**
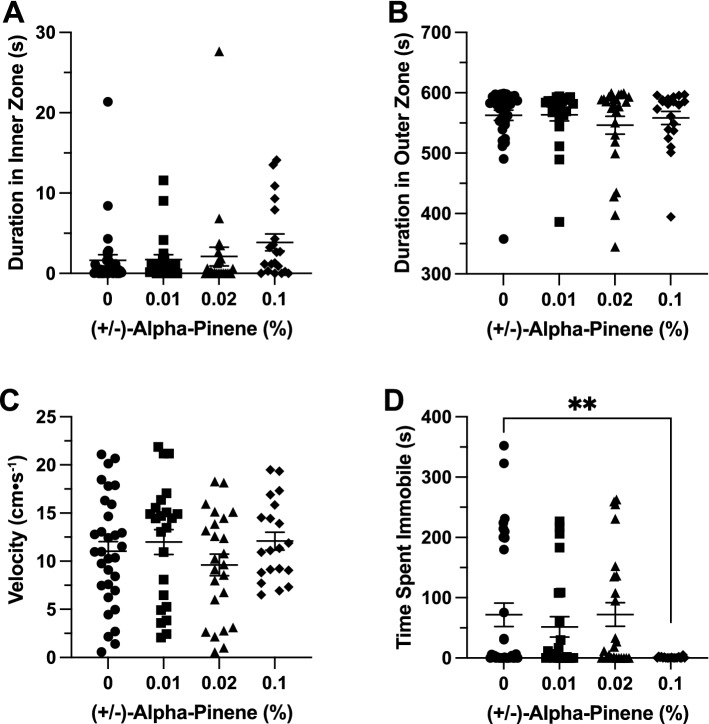
The effects of (+ /−)-alpha-pinene administration in the novel object approach test. Average duration of time fish spent in the (**A**) inner and (**B**) outer ‘thigmotaxis’ zone during the novel object approach test. Fish locomotion was quantified in the novel object approach test by measuring (**C**) swimming velocity and (**D**) time spent immobile. All data are presented as mean ± S.E.M. Significant differences between controls and (+ /−)-alpha-pinene treated groups are indicated by **(*p* < 0.05).

*Time in Zones.* (+ /−)-αPN did not have a significant effect on duration of time spent in the inner zone between groups (F(3, 73.26) = 1.196, *p* = 0.317; Fig. 3A). (+ /−)-αPN did not have a significant effect on duration of time spent in the thigmotaxis zone between groups (H(4) = 0.4499, *p* = 0.93; Fig. 3B).

*Locomotion*. (+ /−)-αPN did not have a significant effect on velocity between groups (F(3, 95) = 1.005, *p* = 0.394; Fig. 3C). (+ /−)-αPN did have a significant effect on duration of time spent immobile between groups (F(3, 75.03) = 3.693, *p* = 0.016). A post-hoc analysis using Dunnett’s multiple comparisons test found a significant decrease in time spent immobile in the 0.1% group (0.88 ± 0.3 s, n = 20, *p* = 0.03) when compared to the control group (71.8 ± 19.5 s, n = 32; Fig. 3D).

### Effects of (−)*-*α-pinene in the open field exploration test

**Figure 4:**
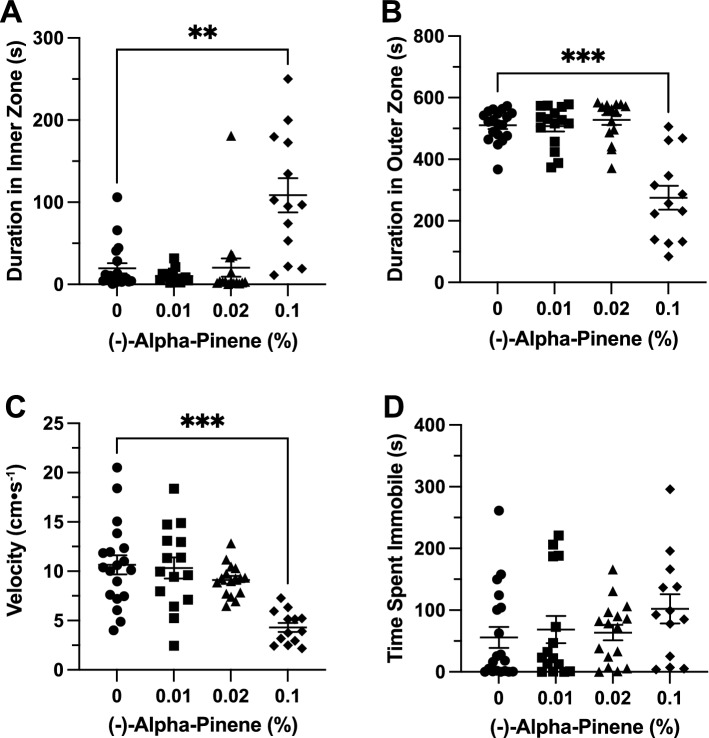
The effects of (−)-alpha-pinene administration in the open field test. Average duration of time fish spent in the (**A**) inner and (**B**) outer ‘thigmotaxis’ zone during the open field test. Fish locomotion was quantified in the open field test by measuring (**C**) swimming velocity and (**D**) time spent immobile. All data are presented as mean ± S.E.M. Significant differences between controls and (−)-alpha-pinene treated groups are indicated by **(*p* < 0.01) and ***(*p* < 0.001).

*Time in Zones*. (−)-αPN had a significant effect on duration of time spent in the inner zone between groups (F(3, 23.28) = 13.36, *p* < 0.001). A post-hoc analysis using Dunnett’s multiple comparisons test found a significant increase in time spent in the inner zone in the 0.1% group (108.6 ± 20.9 s, n = 13, *p* = 0.003) when compared to the control group (19.6 ± 6.3 s, n = 19; Fig. 4A). (−)-αPN had a significant effect on duration of time spent in the thigmotaxis zone between groups (F(3, 26.37) = 25.01, *p* < 0.001). A post-hoc analysis using Dunnett’s multiple comparisons test found a significant decrease in time spent in the thigmotaxis zone in the 0.1% group (275.2 ± 38.76 s, n = 13, *p* < 0.001) when compared to the control group (510.2 ± 11.9 s, n = 19; Fig. 4B).

*Locomotion*. (−)-αPN had a significant effect on velocity between groups (F(3, 59) = 11.18, *p* < 0.001). A post-hoc analysis using Dunnett’s multiple comparisons test indicated significant decreases in velocity between the 0.1% (4.3 ± 0.5 cm s^−1^, n = 13, *p* < 0.001) group when compared to the control group (10.7 ± 0.97 cm s^−1^, n = 19; Fig. 4C). (−)-αPN did not have a significant effect on duration of time spent immobile between groups (H(4) = 4.16, *p* = 0.25; Fig. 4D).

### Effects of (−)*-*α-pinene in the novel object approach test

**Figure 5:**
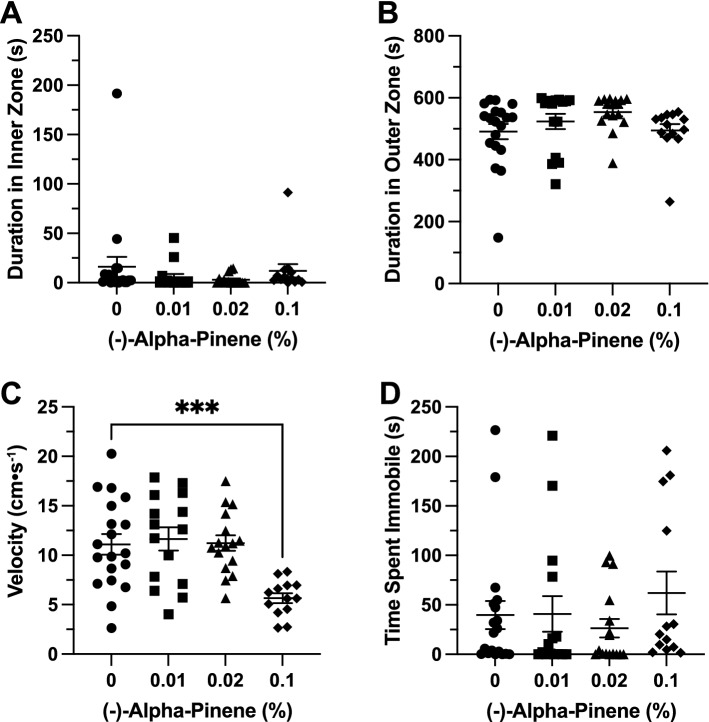
The effects of (−)-alpha-pinene administration in the novel object approach test. Average duration of time fish spent in the (**A**) inner and (**B**) outer ‘thigmotaxis’ zone during the novel object approach test. Fish locomotion was quantified in the novel object approach test by measuring (**C**) swimming velocity and (**D**) time spent immobile. All data are presented as mean ± S.E.M. Significant differences between controls and (−)-alpha-pinene treated groups are indicated by ***(*p* < 0.001).

*Time in Zones*. (−)-αPN did not have a significant effect on duration of time spent in the inner zone between groups (F(3, 32.06) = 0.9235, *p* = 0.441; Fig. 5A). (−)-αPN did not have a significant effect on duration of time spent in the thigmotaxis zone between groups (H(4) = 9.25, *p* = 0.026; Fig. 5B).

*Locomotion*. (−)-αPN did have a significant effect on velocity between groups (F(3, 48.26) = 8.240, *p* < 0.001). A post-hoc analysis using Dunnett’s multiple comparisons test indicated significant decreases in velocity between the 0.1% (5.7 ± 0.5 cm s^−1^, n = 13, *p* < 0.001) group when compared to the control group (11.1 ± 1.0 cm s^−1^, n = 19; Fig. 5C). (−)-αPN did not have a significant effect on duration of time spent immobile between groups (H(4) = 4.294, *p* = 0.231; Fig. 5D).

### Effects of (+)*-*α-pinene in the open field exploration test

**Figure 6:**
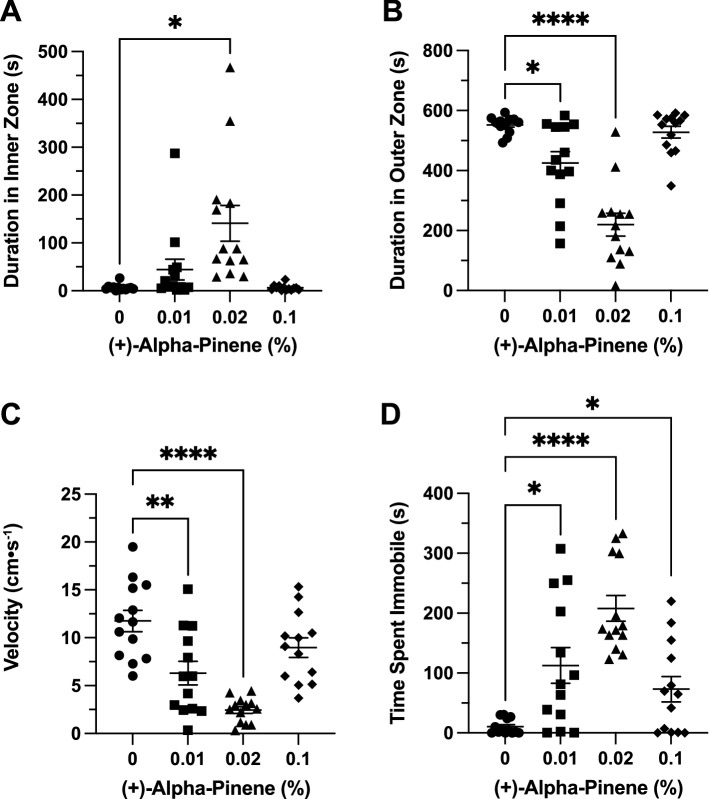
The effects of (+)-alpha-pinene administration in the open field test. Average duration of time fish spent in the (**A**) inner and (**B**) outer ‘thigmotaxis’ zone during the open field test. Fish locomotion was quantified in the open field test by measuring (**C**) swimming velocity and (**D**) time spent immobile. All data are presented as mean ± S.E.M. Significant differences between controls and (+)-alpha-pinene treated groups are indicated by *(*p* < 0.01), **(*p* < 0.001), and ****(*p* < 0.0001).

*Time in Zones*. (+)-αPN had a significant effect on duration of time spent in the inner zone between groups (F(3, 19.45) = 8.657, *p* < 0.001). A post-hoc analysis using Dunnett’s multiple comparisons test found a significant increase in time spent in the inner zone in the 0.02% group (140.9 ± 37.2 s, n = 13, *p* = 0.011) when compared to the control group (6.6 ± 1.8 s, n = 13; Fig. 6A). (+)-αPN had a significant effect on duration of time spent in the thigmotaxis zone between groups (F(3, 30.83) = 27.5, *p* < 0.0001). A post-hoc analysis using Dunnett’s multiple comparisons test found a significant decrease in time spent in the thigmotaxis zone in the 0.01% (425.1 ± 38.1 s, n = 13, *p* = 0.018) and 0.02% (219.6 ± 38.0 s, n = 13, *p* < 0.0001) groups when compared to the control group (552.4 ± 7.7 s, n = 13; Fig. 6B).

*Locomotion*. (+)-αPN had a significant effect on velocity between groups (F(3, 37.48) = 16.05, *p* < 0.0001). A post-hoc analysis using Dunnett’s multiple comparisons test indicated significant decreases in velocity between the 0.01% (6.3 ± 1.2 cm s^−1^, n = 13, *p* = 0.001) and 0.02% (2.5 ± 0.4 cm s^−1^, n = 13, *p* < 0.0001) groups when compared to the control group (11.8 ± 1.1 cm s^−1^, n = 13; Fig. 6C). (+)-αPN had a significant effect on duration of time spent immobile between groups (F(3, 32.63) = 15.15, *p* < 0.0001). A post-hoc analysis using Dunnett’s multiple comparisons test indicated significant increases in immobility between the 0.01% (112.6 ± 29.9 s, n = 13, *p* = 0.015), 0.02% (208.0 ± 21.5 s, n = 13, *p* < 0.0001), and 0.1% (73.02 ± 21.2 s, n = 13, *p* = 0.034) groups when compared to the control group (10.3 ± 3.5 s, n = 13; Fig. 6D).

### Effects of (+)***-***α-pinene in the novel object approach test

**Figure 7:**
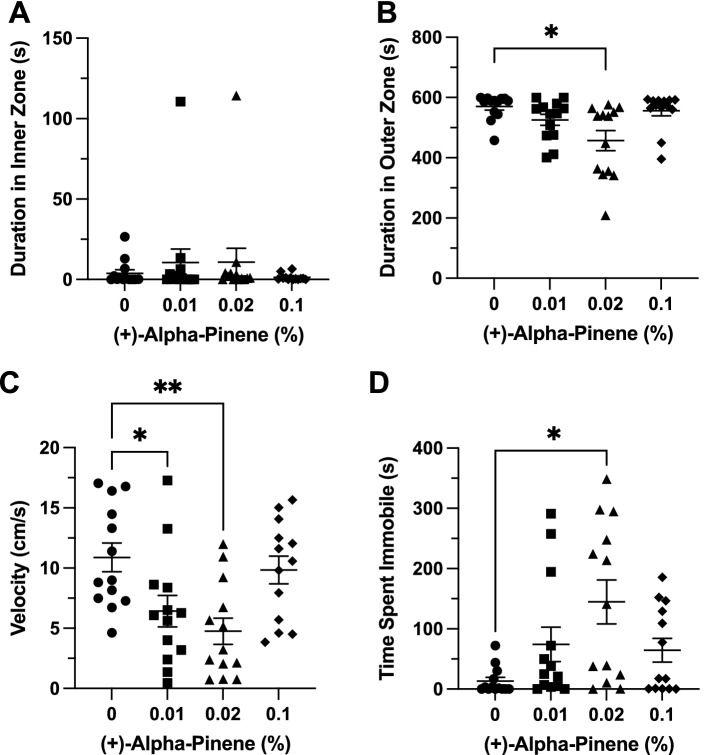
The effects of (+)-alpha-pinene administration in the novel object approach test. Average duration of time fish spent in the (**A**) inner and (**B**) outer ‘thigmotaxis’ zone during the novel object approach test. Fish locomotion was quantified in the novel object approach test by measuring (**C**) swimming velocity and (**D**) time spent immobile. All data are presented as mean ± S.E.M. Significant differences between controls and (+)-alpha-pinene treated groups are indicated by *(*p* < 0.05) and **(*p* < 0.01).

*Time in Zones.* (+)-αPN had no significant effect on duration of time spent in inner zone between groups (F(3, 25.6) = 0.6124, *p* = 0.613; Fig. 7A). (+)-αPN had a significant effect on duration of time spent in the thigmotaxis zone between groups (F(3, 28.96) = 5.379, *p* = 0.005). A post-hoc analysis using Dunnett’s multiple comparisons test found a significant decrease in time spent in the thigmotaxis zone in the 0.02% group (457.1 ± 33.4 s, n = 13, *p* = 0.017) when compared to the control group (570.0 ± 11.5 s, n = 13; Fig. 7B).

*Locomotion*. (+)-αPN had a significant effect on velocity between groups (F(3, 48) = 5.855, *p* = 0.002). A post-hoc analysis using Dunnett’s multiple comparisons test indicated significant decreases in velocity between the 0.01% (6.4 ± 1.3 cm s^−1^, n = 13, *p* = 0.028) and 0.02% (4.8 ± 1.1 cm s^−1^, n = 13, *p* = 0.002) groups when compared to the control group (10.9 ± 1.2 cm s^−1^, n = 13; Fig. 7C). (+)-αPN had a significant effect on duration of time spent immobile between groups (F(3, 30.77) = 4.568, *p* = 0.009). A post-hoc analysis using Dunnett’s multiple comparisons test found a significant increase in immobility in the 0.02% group (144.7 ± 36.4 s, n = 13, *p* < 0.01) when compared to the control group (13.2 ± 6.2 s, n = 13; Fig. 7D).

### Effects of β-caryophyllene in the open field exploration test

**Figure 8:**
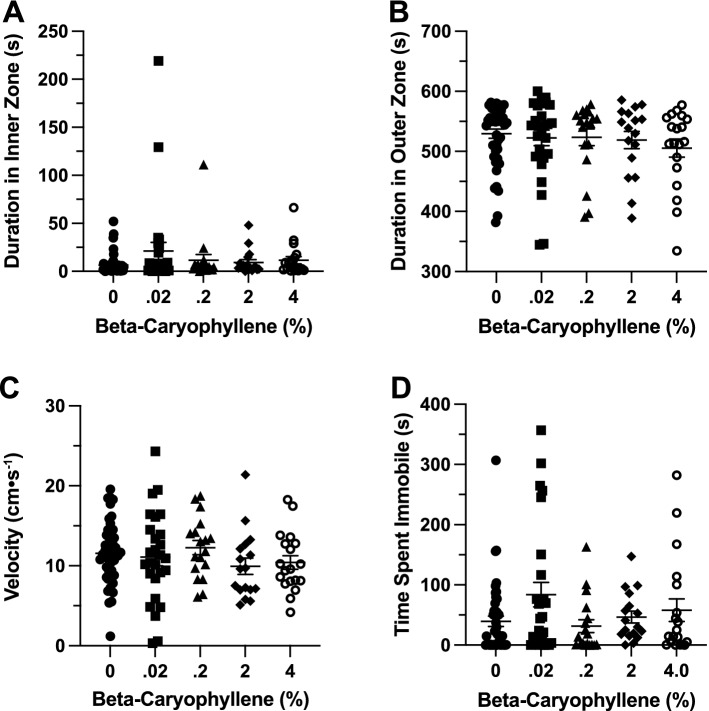
The effects of beta-caryophyllene administration in the open field test. Average duration of time fish spent in the (**A**) inner and (**B**) outer ‘thigmotaxis’ zone during the open field test. Fish locomotion was quantified in the open field test by measuring (**C**) swimming velocity and (**D**) time spent immobile. All data are presented as mean ± S.E.M.

*Time in Zones*. βCP had no significant effect on duration of time spent in inner zone between groups (F(4, 53.64) = 1.337, *p* = 0.268; Fig. 8A). βCP had no significant effect on duration of time spent in thigmotaxis zone between groups (H(5) = 2.412, *p* = 0.66; Fig. 8B).

*Locomotion*. βCP had no significant effect on velocity between groups (H(5) = 5.083, *p* = 0.279; Fig. 8C). βCP also had no significant effect on duration of time spent immobile between groups (F(4, 75.85) = 2.150, *p* = 0.083; Fig. 8D).

### Effects of β-caryophyllene in the novel object approach test

**Figure 9:**
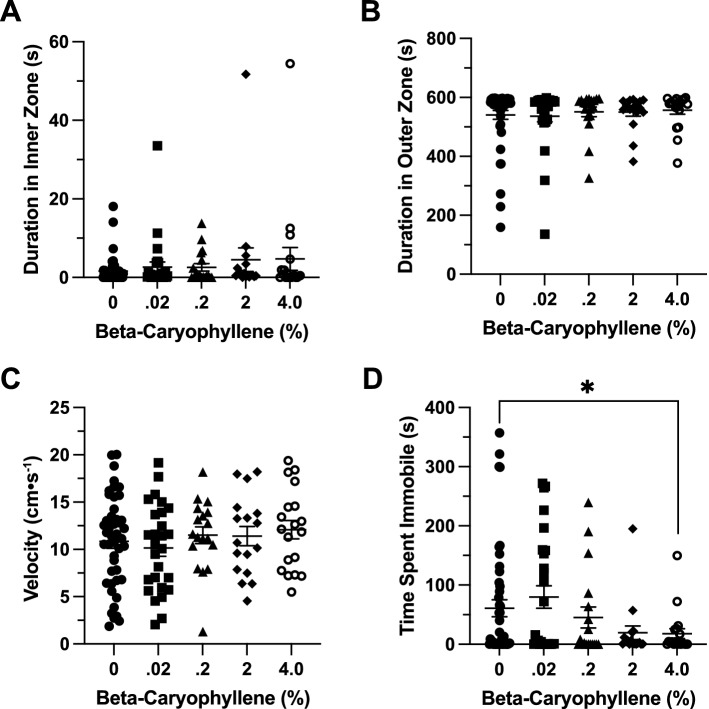
The effects of beta-caryophyllene administration in the novel object approach test. Average duration of time fish spent in the (**A**) inner and (**B**) outer ‘thigmotaxis’ zone during the novel object approach test. Fish locomotion was quantified in the novel object approach test by measuring (**C**) swimming velocity and (**D**) time spent immobile. All data are presented as mean ± S.E.M. Significant differences between controls and beta-caryophyllene treated groups are indicated by *(*p* < 0.05).

*Time in Zones*. βCP had no significant effect on duration of time spent in inner zone between groups (F(4, 48.69) = 0.5634, *p* = 0.69; Fig. 9A). βCP had no significant effect on duration of time spent in thigmotaxis zone between groups (F(4, 110.9) = 0.2597, *p* = 0.903; Fig. 9B).

*Locomotion*. βCP had no significant effect on velocity between groups (H(5) = 2.331, *p* = 0.675; Fig. 9C). βCP did have a significant effect on duration of time spent immobile between groups (F(4, 97.77) = 3.033, *p* = 0.021). A post-hoc analysis using Dunnett’s multiple comparisons test found a significant decrease in immobility in the 4.0% group (17.9 ± 8.4 s, n = 19, *p* < 0.05) when compared to the control group (60.7 ± 14.3 s, n = 45; Fig. 9D).

## Discussion

This study investigated the anxiolytic and locomotor effects of two commonly found cannabis terpenes in North American cannabis strains, α-pinene and its optical (+) and (−) enantiomers, and β-caryophyllene, using the open field exploration test and the novel object approach test. While (+ /−)-αPN showed no effects on either anxiety variables measured in both tests, both (+) and (−) αPN enantiomers decreased anxiety-like behaviour in the open field test by significantly increasing time spent in the inner zone and decreasing time spent in the thigmotaxis zone. In both (+) and (−) groups, however, significant effects on behaviour were decreased or eliminated with the introduction of a novel object. Interestingly, (−)-αPN demonstrated strong anxiolytic effects at our highest (0.1%) treatment group. While (+)-αPN demonstrated anxiolytic effects only at the low (0.01%) and moderate (0.02%) treatment groups. (+ /−)-αPN had no effect on velocity while significantly decreasing immobility in both open field and novel object approach tests. Significant decreases in velocity and increases in immobility were found in both the low and moderate (+)-αPN doses, however, in both open field and novel object approach, (−)-αPN significantly decreased velocity at our highest dose but had no effect on immobility. βCP had no effect on either anxiety measure or velocity across both behavioural tests. Interestingly, however, βCP did significantly decrease immobility in the novel object approach test.

Increased swimming velocity and immobility have been suggested to indicate heightened levels of anxiety in previous studies with zebrafish^24,32–41^. However, measures of velocity and immobility have not consistently corresponded to main effect measures of anxiety-like behaviour across most zebrafish behavioural paradigms^42^. This suggests locomotor responses vary depending on the test used. For example, increased swimming velocity may correspond to avoidance behaviour and heightened anxiety, or more risky behaviour (increased exploration) and decreased anxiety. Similarly, increased immobility may suggest a freezing response associated with anxiety, or lack of movement associated with sedation and a relaxed state. Furthermore, decreased velocity may also suggest a sedative response rather than an anxiolytic response. Therefore, it is necessary to validate the reliability of these measures in relation to zebrafish anxiety-like behaviour and the behavioural test being used^25^.

Fish in both (+) and (−) αPN enantiomer groups in the open field and novel object approach tests demonstrated a significant reduction in swimming velocity. However, fish in the (+) enantiomer group had a significant difference in immobility, whereas the (−) enantiomer group had no change in immobility. Therefore, the decreased velocity and increased immobility induced by (+)-αPN suggests a strong sedative effect, while (−)-αPN has only minor sedative action. Further testing with a higher (−)-αPN dose is required to determine whether (−)-αPN will show a similar non-linear, sedative effect at higher doses. Interestingly, counter to the effect on immobility observed in the (+)-αPN group, (+ /−)-αPN decreased immobility in both open field and novel object approach tests. This finding demonstrates (+ /−)-⍺-pinene and each of its (+) and (−) isomeric compounds have differential anxiety-like and locomotor behavioural effects at different doses.

βCP had no effect across all variables of interest in the open field test or novel object approach test in any of the treatment groups when compared to the control, aside from a modest decrease in immobility in the novel object approach test in the highest dose used (4.0%). Several studies using mice have reported βCP to display an anxiolytic effect at higher doses^13,14,17,18^. Due to the novel nature of this study, no dose parameters for βCP have been validated to reliably produce a behavioural alteration in zebrafish models, therefore, further pilot testing is needed. Our results show a potential dose-dependent downward trend in anxiety levels, which suggests that a higher dose may be effective. However, due to the low aqueous solubility of the compound it was not possible to increase the dose level beyond what was employed here. In addition to poor water solubility, previous pharmacokinetic studies have noted βCP to be highly volatile and sensitive to light, oxygen, humidity, and high temperatures^43^, which may inhibit bioavailability of the terpene. Therefore, the observed weak or non-effect of this compound could be attributed to a low absorption rate, as well as metabolism and excretion rate. Further behavioural testing is required to assess whether a higher dose or different delivery method will elicit a significant response.

Phytocannabinoids found in cannabis are exogenous ligands that act on the cannabinoid receptors found in most species of the *animalia* kingdom^44^. For example, both phytocannabinoids, ∆^9^-THC and CBD, bind to CB_1_ and CB_2_ receptors in the endocannabinoid system^45,46^. Thus, it is feasible that the terpene compounds found in cannabis plants may also act on cannabinoid receptors. While ∆^9^-THC and CBD are known to produce anxiolytic and other therapeutic effects, it is unknown whether this may be due to the modulatory effects of other cannabis constituents such as terpene compounds^47^. Russo^1^ demonstrated the ‘entourage effect’ showing how terpenes may actually alter the effects of phytocannabinoids. However, recent studies exploring the entourage effect did not detect CB receptor-mediated modulations of terpenes on the effects of THC or CBD^48–50^. With recent studies demonstrating terpene compounds to have similar effects as THC and CBD on endocannabinoid receptors, it is important to test their mechanisms of action and medicinal properties in isolation from other properties of the cannabis plant^47,50^.

The endocannabinoid system, specifically cannabinoid CB_1_ and CB_2_ receptors, have been shown to regulate mood and anxiety disorders^51–53^. CB_1_ receptors are distributed across the central nervous system (CNS) and are known to decrease the release of dopamine, norepinephrine, glutamate, and serotonin, while CB_2_ is said to be associated with the immune system^44,54,55^. Interestingly, several studies have shown that the effects of βCP are mediated through the selective binding to CB_2_ receptors because a CB_2_ antagonist eliminated its effects^13,16,56^. However, other studies have shown that βCP may not act on endocannabinoid receptors^48,49^, thus its mechanism of action in the brain is unclear. If βCP acts on CB_2_ receptor sites this may contribute to its potential to have anxiolytic and antidepressant effects in animals^5,13^. Bahi and colleagues^13^ describe that previously, CB_2_ receptors were thought to be absent in the brain, but have now been identified in the CNS and play a role in anxiety and depressive-related disorders. Although βCP’s mechanism of action has not been clearly defined, it has shown potential to act on CB_2_ receptors^13,16,56^. Bahi and colleagues^13^ postulate that drug alternatives acting through CB_2_ receptors could become novel pharmacological therapies in the treatment of anxiety and mood disorders.

Molecular research demonstrates that both the endocannabinoid and GABAergic systems are associated with the pathophysiology of anxiety and related disorders^57,58^. While αPN has not been shown to have an affinity for CB_1_ or CB_2_ receptors^12^, it has been demonstrated to interact with the GABA_A_ receptor complex to prolong GABAergic synaptic transmission^21,59^, which is likely to contribute to its potential sedative and anxiolytic effects^11,20,30^. ⍺-pinene has been shown to target certain GABA neurons resulting in a range of psychophysiological effects^21^. Specifically, ⍺-pinene acts on GABA neurons by generating a presynaptic response to signal neurons to inhibit GABA reuptake transporters which can alleviate symptoms of anxiety and insomnia^46^.

Studies have identified GABA_A_ receptors in zebrafish and researchers have found they possess a conserved GABAergic system^60–63^. Zebrafish have also been shown to express all of the major endocannabinoid-related genes, such as, CB_1_ and CB_2_^64,65^, and are a relatively efficient experimental model for the anxiolytic effects of cannabinoids and terpenes. Therefore, future studies exploring the mechanisms of action with terpene administration along with CB_1_ and CB_2_ antagonists, and selective binding of βCP and ⍺PN on zebrafish receptor sites could provide substantial evidence of the potential interaction of terpenes and cannabinoids.

## Conclusion

(+ /−)-⍺-pinene and its (+) and (−) enantiomers each demonstrated varying effects on zebrafish anxiety-like and locomotor behaviours. (+ /−)-⍺PN had no effects on the anxiety measures, time spent in zones, but had a modest effect on time spent immobile in the highest dose (0.1%). The highest dose of (−)-⍺PN showed a modest effect on time spent in zones and zebrafish swimming velocity but not immobility, while (+)-⍺PN showed a strong effect across all variables, primarily in the low and moderate doses. In both groups, anxiolytic effects in the open field test were reduced or eliminated with the introduction of a novel object. These results demonstrate the differential dose-dependent effect of (+ /−)-⍺-pinene and each of its (+) and (−) isomeric compounds. β-caryophyllene had little to no effect across tests on any of the variables analyzed in this study, therefore, further testing is required to determine if a higher dose would yield significant results.

## Supplementary Information

Supplementary Information 1.

Supplementary Information 2.

## Data Availability

Available upon request. Correspondence and requests for materials should be addressed to T.J.H. or A.J. [JohnsonA254@mymacewan.ca]. Analyzed data from Noldus EthoVision XT tracking software is available in the electronic supplementary material.
