# Supplementary material for: Effects of super-class cannabis terpenes beta-caryophyllene and alpha-pinene on zebrafish behavioural biomarkers
Source: Sci Rep. 2022 Oct 14;12:17250. doi: 10.1038/s41598-022-21552-2 (PMC9568608; doi:10.1038/s41598-022-21552-2)
Supplement: Supplementary file 1 — Supplementary Information 1. [file 41598_2022_21552_MOESM1_ESM.docx]

Supplementary Table 1

*Statistical Results*

| Treatment | Test | Measure | Test Statistic | P-Value | Sig.^#^ |
| --- | --- | --- | --- | --- | --- |
| (+/-)-αPN | Open Field | Inner Zone | F(3, 59.97) = 2.061 | p = 0.115 | ns |
|  |  | Outer Zone | F(3, 56.39) = 2.679 | p = 0.056 | ns |
|  |  | Velocity | F(3, 95) = 0.417 | p = 0.741 | ns |
|  |  | Immobility | F(3, 64.29) = 2.780 | p = 0.048 | ** |
|  | Novel Object | Inner Zone | F(3, 73.26) = 1.196 | p = 0.317 | ns |
|  |  | Outer Zone | H(4) = 0.4499 | p = 0.93 | ns |
|  |  | Velocity | F(3, 95) = 1.005 | p = 0.394 | ns |
|  |  | Immobility | F(3, 75.03) = 0.016 | p = 0.016 | ** |
| (-)-αPN | Open Field | Inner Zone | F(3, 23.28) = 13.36 | p < 0.001 | ** |
|  |  | Outer Zone | F(3, 26.37) = 25.01 | p < 0.001 | *** |
|  |  | Velocity | F(3, 59) = 11.18 | p < 0.001 | *** |
|  |  | Immobility | H(4) = 4.16 | p = 0.25 | ns |
|  | Novel Object | Inner Zone | F(3, 32.06) = 0.9235 | p = 0.0441 | ns |
|  |  | Outer Zone | H(4) = 9.25 | p = 0.026 | ns |
|  |  | Velocity | F(3, 48.26) = 8.240 | p < 0.001 | *** |
|  |  | Immobility | H(4) = 4.294 | p = 0.231 | ns |
| (+)-αPN | Open Field | Inner Zone | F(3, 19.45) = 8.657 | p < 0.001 | * |
|  |  | Outer Zone | F(3, 30.83) = 27.5 | p < 0.0001 | *, **** |
|  |  | Velocity | F(3, 37.48) = 16.05 | p < 0.0001 | **, **** |
|  |  | Immobility | F(3, 32.63) = 15.15 | p < 0.0001 | *, **** |
|  | Novel Object | Inner Zone | F(3, 25.6) = 0.6124 | p = 0.613 | ns |
|  |  | Outer Zone | F(3, 28.96) = 5.379 | p = 0.005 | * |
|  |  | Velocity | F(3, 48) = 5.855 | p = 0.002 | *, ** |
|  |  | Immobility | F(3, 30.77) = 4.568 | p = 0.009 | * |
| βCP | Open Field | Inner Zone | F(4, 53.64) = 1.337 | p = 0.268 | ns |
|  |  | Outer Zone | H(5) = 2.412 | p = 0.66 | ns |
|  |  | Velocity | H(5) = 5.083 | p = 0.279 | ns |
|  |  | Immobility | F(4, 75.85) = 2.150 | p = 0.083 | ns |
|  | Novel Object | Inner Zone | F(4, 48.69) = 0.5634 | p = 0.069 | ns |
|  |  | Outer Zone | F(4, 110.9) = 0.2597 | p = 0.903 | ns |
|  |  | Velocity | H(5) = 2.331 | p = 0.675 | ns |
|  |  | Immobility | F(4, 97.77) = 3.033 | p = 0.021 | * |

*Note.* Significant differences between controls and treatment groups are indicated by *(P < 0.05), **(P < 0.01), ***(P < 0.001) and ****(P < 0.0001).

^#^ Please refer to figures for group comparisons.
